# Carboxylated-xyloglucan and peptide amphiphile co-assembly in wound healing

**DOI:** 10.1093/rb/rbab040

**Published:** 2021-08-11

**Authors:** Alessia Ajovalasit, Carlos Redondo-Gómez, Maria Antonietta Sabatino, Babatunde O Okesola, Kristin Braun, Alvaro Mata, Clelia Dispenza

**Affiliations:** 1 Dipartimento di Ingegneria (DI), Università degli Studi di Palermo, Viale delle Scienze, Edificio 6, Palermo 90128, Italy; 2 School of Engineering & Materials Science, Queen Mary University of London, London E1 4NS, UK; 3 Institute of Bioengineering, Queen Mary University of London, London E1 4NS, UK; 4 Blizard Institute, Barts and The London School of Medicine and Dentistry, The Blizard Building, 4 Newark Street, London E1 2AT, UK; 5 School of Pharmacy, University of Nottingham, Nottingham NG7 2RD, UK; 6 Department of Chemical and Environmental Engineering, University of Nottingham, Nottingham NG7 2RD, UK; 7 Biodiscovery Institute, University of Nottingham, Nottingham, NG7 2RD, UK; 8 Istituto di Biofisica (IBF), Consiglio Nazionale Delle Ricerche (CNR), Via U. La Malfa 153, Palermo 90146, Italy

**Keywords:** self-assembly, peptide nanofiber, hydrogel, wound healing, skin tissue engineering

## Abstract

Hydrogel wound dressings can play critical roles in wound healing protecting the wound from trauma or contamination and providing an ideal environment to support the growth of endogenous cells and promote wound closure. This work presents a self-assembling hydrogel dressing that can assist the wound repair process mimicking the hierarchical structure of skin extracellular matrix. To this aim, the co-assembly behaviour of a carboxylated variant of xyloglucan (CXG) with a peptide amphiphile (PA-H3) has been investigated to generate hierarchical constructs with tuneable molecular composition, structure, and properties. Transmission electron microscopy and circular dichroism at a low concentration shows that CXG and PA-H3 co-assemble into nanofibres by hydrophobic and electrostatic interactions and further aggregate into nanofibre bundles and networks. At a higher concentration, CXG and PA-H3 yield hydrogels that have been characterized for their morphology by scanning electron microscopy and for the mechanical properties by small-amplitude oscillatory shear rheological measurements and compression tests at different CXG/PA-H3 ratios. A preliminary biological evaluation has been carried out both *in vitro* with HaCat cells and *in vivo* in a mouse model.

## Introduction

In the last decades, the field of skin tissue engineering has made significant advancements towards faster wound healing and restoration of skin structure, functionality and aesthetics. Various types of skin substitutes have been proposed to support skin regeneration: scaffolds designed to provide the ideal environment for recruiting the patient’s own cells [1], temporary skin substitutes containing allogeneic skin cells [2], or permanent substitutes containing autologous cells such as fibroblasts, keratinocytes or stem cells [3, 4]. The combination of autologous cells with biodegradable scaffolds is probably the most popular strategy. This approach offers the possibility of partially controlling the colonization of the scaffold *in vitro* prior to any implantation procedure or, indeed, to proceed immediately with the implantation post-seeding. It also limits the risks of an immune response.

Hydrogels are extensively investigated as scaffolds for their ability to mimic the extracellular matrix (ECM) in providing support and biochemical cues to the cells, enhance cell adhesion and accelerate structural repair [5–9]. Hydrogels are also commercially used as wound dressings, for their ability to supply water and molecular oxygen to the wound bed, protect the wound from impact and abrasion, and absorb and lock exudates away from the wound bed reducing maceration. With respect to gauze materials, hydrogels are softer and more conformable; they allow more intimate contact with the wound surface, reducing ‘dead space’ where bacteria may reside, and can be removed without causing damage to the newly formed skin. Hydrogel dressings can also be loaded with antimicrobial compounds to treat infected wounds, or with growth factors to accelerate the healing process [10–15]. Moreover, degradable hydrogels are used as resorbable dressings to combine scaffolding functions with the haemostatic and protective functions typical of wound dressings [16].

Molecular self-assembly, the process by which individual components spontaneously assemble through non-covalent interactions into well-defined and reproducible higher ordered structures, is an attractive methodology to produce hydrogel dressings because of the capacity to display desirable ECM signals in a controlled manner [17]. This approach avoids the need for purification steps from catalysts and unreacted crosslinking agents [18]. Peptide amphiphiles (PAs) are a particularly promising family of self-assembling peptides [19], characterized by a lipid hydrophobic group, a β-sheet forming peptide and a hydrophilic peptidic segment. These molecules recreate the nanofibrous structure of the natural ECM and can easily be designed to display terminal bioactive segments to guide cell behaviours [20], tailor structural properties [21] and interact with other macromolecules [22, 23]. Taking advantage of the modular nature of PAs and the opportunities of multicomponent self-assembly [24], we have developed co-assembling strategies to generate PA-macromolecule composite materials with improved mechanical properties [25], structural hierarchy [26, 27] and the capacity to recreate complex biological environments [28, 29].

Building on this supramolecular toolkit, here we report on the co-assembly of a cationic PA with a carboxylated variant of the tamarind seed xyloglucan. Xyloglucan (XG) is a non-ionic, water-soluble polysaccharide composed of a *β*-(1,4)-D-glucan backbone, partially substituted by *α*-(1,6)-linked xylose unit. Some of the xylose residues are β-D-galactosylated at the O-2 (for the chemical structure see Supplementary Fig. S1) [30]. XG is FDA approved for use as food additive, due to its ability to act as thickener and stabilizing agent, and widely investigated as pharmaceutical excipient [31–33]. Moreover, it has also been demonstrated to have intrinsic anti-inflammatory properties and potential beneficial effects in skin re-epithelization and remodelling [34, 35]. Thermo-reversible hydrogels can be obtained from a partially degalactosylated variant of XG (dXG). Injectable, *insitu* gelling dXG-based formulations, loaded with adipose stem cell spheroids, have been demonstrated to enhance cell viability and stemness properties [36, 37]. Hydro-alcoholic physical hydrogels based on XG or XG/polyvinyl alcohol blends have been evaluated as wound dressings for their favourable mechanical properties and biocompatibility [38–40]. These hydrogels have also been integrated with inexpensive, lightweight, ultra-high frequency radio-frequency identification (UHF RFID) sensor tags that can battery-less monitor temperature and moisture level of the dressing as well as wireless transmit the measured data to an off-body reader [39].

Co-assembly of cationic PAs with other anionic polysaccharides has been reported to generate delicate fibrous structures in the form of sacs or membranes, which are structurally advantageous for skin applications but have a limiting factor in their relatively poor mechanical properties [22, 27].

By co-assembling PAs with XG, we aim to develop more robust and resilient hydrogels that still exhibit fibrous, skin ECM-like architecture and can demonstrate adequate durability to survive prolonged exposure to biological fluids. Moreover, we can expect good cellular adhesiveness owing to the galactose residues present in the CXG sidechains that can interact with galectin receptors involved in the modulation of cell-matrix interactions and wound healing [41, 42].

In order to provide XG with electric charges, carboxylation was pursued via a TEMPO-mediated oxidation reaction [43–46]. The degree of carboxylation, related changes in molecular weight and surface charge density of the oxidized XG variant (CXG) were investigated via infrared spectroscopy and acid-base titration, gel permeation chromatography, static light scattering and ζ-potential measurements. A cationic (at physiologic pH) PA, characterized by the hydrophobic palmitoyl chain, the -(Val)_3_(Ala)_3_- β-sheet forming motif and the (His)_3_ amino terminal acid sequence, was synthetized according to an established procedure and named after PA-H3 (for the chemical structure, see Supplementary Fig. S2) [47]. To the best of our knowledge, PA-H3 has never been used before in co-assembly with polysaccharides.

As a preliminary screening, the best co-assembly procedure and the most convenient pH of both PA-H3 and CXG solutions were identified by visual inspection of the hydrogels obtained mixing the two solutions at 1:1 volume ratio and 1%w concentration. Then, the co-assembly structures obtained by mixing the PA-H3 solution with different volumes of CXG solution were investigated at low concentration, by circular dichroism (CD) and transmission electron microscopy (TEM), to have an insight into the co-assembly mechanism. The macroscopic hydrogels obtained at higher concentration were characterized for their morphological and mechanical properties by scanning electron microscopy (SEM), dynamic mechanical rheological analysis, and uniaxial quasi-static compression tests. Selected hydrogels were also evaluated in terms of fibroblast cell viability and adhesion, and ability to promote wound closure *in vivo.*

## Materials and methods

### Materials

XG was purchased from Megazyme International (Ireland). Sugar composition is xylose 34%w; glucose 45%w; galactose 17%w; arabinose and other sugars 4%w, as provided by the supplier. Fmoc-protected amino acids and MBHA Rink Amide resin were purchased from Novabiochem Corporation (UK). 1-hydroxybenzotriazole hydrate (HOBT) was purchased from Carbosynth Limited. Palmitic acid was purchased from Calbiochem. Piperidine and triisopropylsilane (TIS) was purchased from Alpha Aesar. N,N′-diisopropylcarbodiimide (DIC), 2,2,6,6-tetramethyl-1-piperidinyloxy (TEMPO), 4-(2-hydroxyethyl)piperazine-1-ethanesulfonic acid (HEPES), polydimethylsiloxane (PDMS, SYLGARD^®^ 184 kit) acid, the Kaiser test kit (phenol 80% in ethanol, KCN in H_2_O/pyridine and ninhydrin 6% in ethanol), trifluoracetic acid (TFA), sodium bromide (NaBr), sodium hypochlorite solution, sodium borohydride (NaBH_4_), dichloromethane (DCM), N,N-dimethylformamide (DMF), diethyl ether and ethanol were all purchased from Sigma Aldrich. Dulbecco’s modified Eagle’s medium (DMEM), Hank’s balanced salt solution (HBSS), penicillin/streptomycin (P/S), foetal bovine serum (FBS) were obtained from Gibco (Life Technologies). All the reagents were employed as received.

### Methods

#### Synthesis and characterization of CXG

In a typical carboxylation reaction, 150 ml of 0.2%w XG aqueous dispersion was placed in an ice bath and deaerated with gaseous nitrogen for 30 min while stirring. To this system, 3 mg of TEMPO, 12.5 mg NaBr and 720 μl of 15%w sodium hypochlorite aqueous solution were added. During the oxidation process, small volumes of 0.1 M NaOH were added to maintain the pH at about 9. The reaction was stopped after 4 h, by adding 22.8 mg of NaBH_4_. The polymer was then recovered by precipitation in cold ethanol followed by freeze dying.

CXG was characterized by infrared spectroscopy (FTIR) with Spectrum Two FTIR spectrometer, (Perkin Elmer) in its fully protonated form. Spectra were collected by accumulation of 32 scans between 4000 and 450 cm^−1^, with a resolution of 4 cm^−1^. All spectra have been normalized with respect to the peak correspondent to the stretching of methylene groups (2956 cm^−1^). The quantitative estimation of carboxyl groups in CXG was performed by acid–base titration.

The average weight molecular weight (Mw) of XG and CXG were assessed through Zimm plot analysis of static light scattering (SLS) data from measurements carried out at different angles and polymer concentrations after filtration with 0.45 µm syringe filters, using a Brookhaven BI-9000 correlator and a 50 mW He–Ne laser (MellesGriot) tuned at λ = 632.8 nm and BI200-SM goniometer. The measurements were performed at 25 ± 0.1°C. The refractive index increment (dn/dc) of XG and CXG in solution, measured by using a Brookhaven Instruments differential refractometer at λ = 620 nm, were of 0.155 ± 0.005 ml/g and 0.150 ± 0.004 ml/g, respectively. The electric surface charge density of CXG in HEPES at pH 7.4 and in water at various pHs was measured in triplicate, by laser Doppler velocimetry using a Zetasizer Instrument (NANO-ZS ZEN3600, Malvern Instruments, UK) at 25°C.

Distributions of hydrodynamic volumes were determined by gel filtration chromatography (GFC) conducted using a Shodex SB HQ (804 and 806) columns coupled with an Agilent 1260 Infinity HPLC with a refractive index detector. Prior to injection in the column, the polymer aqueous solutions were filtered with 0.8 µm cellulose acetate (Millipore) syringe filters. Chromatograms of the samples were compared with chromatograms of pullulan standard (Sigma Aldrich) solutions.

#### Synthesis and characterization of PA-H3

PA-H3 was prepared on a 0.5 mmol scale using MBHA Rink Amide resin, following a modified variant of a previously published protocol [48]. All amino acid couplings were performed using four equivalents (4 mmol) of Fmoc-protected amino acids, four equivalents of HOBT and six equivalents of DIC dissolved in DMF. Fmoc groups were removed with a 20%v piperidine solution in DMF and the Kaiser test kit was used to confirm Fmoc removal. A palmitoyl tail was attached using four equivalents of palmitic acid, four equivalents of HOBT, and six equivalents of DIC in DMF/DMC (3:2). The reaction was carried out overnight. Then, the resin was washed several times with DCM and DMF, and PA-H3 was cleaved from the resin by washing with TFA/TIS/water (95:2.5:2.5) for 3 h at room temperature. The solution was then collected by filtration and all washings were combined and roto-evaporated to remove residual TFA. The crude PA-H3 solution was precipitated by addition of an excess of cold (−20°C) diethyl ether. The precipitate was collected by centrifugation, washed several times with cold diethyl ether and dried overnight under vacuum.

For the purification, the crude PA-H3 was dissolved in water and freeze-dried before preparative high-performance liquid chromatography (HPLC) purification. The solid was then dissolved in deionized water at 5 mg/ml and purified using a 2545 binary gradient preparative High-Performance Liquid Chromatographer (Waters, USA) with a 2489 UV/Visible detector (Waters, USA) using a C18 column (Atlantis Prep OBD T3 Column, Waters, USA) and a water/acetonitrile (0.1% TFA) gradient. Purified fractions of were finally lyophilized and stored as dry powder. The molecular mass of the product was confirmed by electrospray ionization mass spectrometry (ESI-MS, Thermo LXQ, Thermo Scientific, USA). The electric surface charge density of PA-H3 in HEPES at pH 7.4 and in water at various pHs was measured in triplicate, by laser Doppler velocimetry using a Zetasizer Instrument (NANO-ZS ZEN3600, Malvern Instruments, UK).

#### Co-assembled CXG/PA hydrogel preparation

Hydrogels were initially prepared by placing in contact a given volume of 1%w CXG in isotonic 10 mM HEPES buffer solution (HEPES buffer) with the same volume of HEPES buffer containing 1% w of PA-H3. Different contacting methodologies were assessed: (i) side-by-side contacting; (ii) dropping PA-H3 solution on CXG solution and vice versa; (iii) injecting PA-H3 solution inside CXG solution and vice versa (Supplementary Fig. S3). The solutions were deposited on PDMS coated surfaces and incubated at 28°C and 38% relative humidity for 24 h before visual inspection.

With the best performing contacting methodology (PA-H3 solution injection inside CXG solution), the influence of pH of the two solutions in the range 4–8 was investigated.

Hydrogels were also prepared at different volume ratios (1:1, 2:1, 3:1 and 5:1) between 1%w CXG/HEPES buffer at pH 7–8 and 1%w PA-H3/HEPES buffer at pH 4–5. The systems are coded as CXG_PA-H3 x: y, where x: y is the volume ratio of the two solutions. For comparison, an only PA-H3-containing hydrogel was prepared injecting the PA-H3/HEPES buffer at pH 4–5 inside an equal volume of isotonic PBS. Hydrogel formation was visually captured using a Leica MZ 12-5 microscope (10x).

#### Characterization of co-assembled CXG/PA-H3 systems at low concentration

CXG and PA-H3 solutions were prepared in HEPES buffer at 0.01%w. The pH of the CXG solution was adjusted to 7–8, while the pH of PA-H3 solution was adjusted to 4–5. The solutions of were mixed at different volume ratios (1:1, 2:1, 3:1 and 5:1). Samples were left equilibrate for circa 15 min before circular dichroism measurements. CD spectra were acquired between 190 and 260 nm with a step size of 0.5 nm, under a constant flow of nitrogen at constant pressure of 0.7 MPa and temperature of 25°C, with a Pistar-180 spectropolarimeter (Applied Photophysics, Surrey, UK). Spectra are obtained by averaging three consecutive measurements.

For transmission electron microscopy analysis, CXG and PA-H3 solutions (0.05%w in HEPES buffer) were freshly mixed at different volume ratios (1:1, 2:1, 3:1 and 5:1), then casted onto plasma-etched holey carbon-coated copper grids (Agar Scientific, Stansted, UK) and let stand for 5 min. Excess was blotted out using filter paper before incubation with 2%w uranyl acetate for 30 s. Grids were then washed with ultrapure water for 30 s and air dried for 24 h at room temperature. Bright-field TEM imaging was performed on a JEOL 1230 Transmission Electron Microscope operated at an acceleration voltage of 80 kV. All the images were recorded by a Morada CCD camera (Image Systems).

#### Co-assembled CXG/PA-H3 hydrogels characterization

Hydrogels were characterized for their morphology by scanning electron microscopy after removal of salts by repeated washings with MilliQ water, freezing in liquid nitrogen and freeze-drying. Samples were mounted on aluminium stabs, coated with a gold layer (JEOL JFC-1300 coater) for 90 s at 30 mA and imaged with a Field Emission Scanning Electron Microscope (FESEM-JEOL) at an accelerating voltage of 10 kV.

The mechanical properties of the hydrogels were evaluated by small angle oscillatory shear measurements and compression tests. In particular, TA Instruments AR-G2 rheometer was used to perform oscillatory frequency sweeps with a parallel plate geometry (8 mm diameter) in the range of angular frequencies between 1 and 100 rad/sec, using a fixed strain of 0.5% and at 25°C. The chosen strain value was within the linear viscoelastic region of all systems, as previously determined by strain sweep tests performed at 1 Hz frequency. Compression tests were carried out using an Instron 5967 testing machine on hydrogels kept in DMEM complete medium. A preload of 0.5 mN was used to contact the sample and determine the gauge length. Samples were compressed to 20% strain at a rate of 1% s^−^^1^ and maintained at this deformation level for 120 s. The hydrogel shape was approximated to a sphere. 1%w agarose hydrogel spheres were used for comparison. Compression moduli reported are averaged on eight measurements [49].

### Biological evaluations

#### Cell culture conditions

Cell culture experiments were conducted with HaCat keratinocytes cell line. HaCat were cultured with DMEM medium supplemented with 10%v/v FBS, 1%w/v penicillin and 1%w/v streptomycin. Cells were maintained in a humidified 5% CO_2_ atmosphere at 37.0 ± 0.1°C.

#### Cell viability assays

Hydrogels were prepared under sterile conditions and washed three times with DMEM complete medium before the experiments. HaCat cells were seeded on top of the hydrogels (10 000 cells in a 96 well plate). After seeding, samples were put under agitation for 30 min at 200 rpm and then cultured for maximum 7 days. After 2 days and 7 days, the cells were stained with LIVE/DEAD^®^ ThermoFisher reagent and incubated for 30 min at room temperature before analysis with Leica TCS SP2 confocal microscope. Cell counting was performed via processing of confocal images using the software imageJ [50].

#### Cell incorporation

For cell incorporation experiments, HaCat cells were suspended in 1%w PA-H3/HEPES buffer. Hydrogels were rapidly formed by injection of the PA-H3 solution containing 10 000 cells into 1%w CXG/HEPES buffer. After 10 min, DMEM complete medium was added and the constructs were cultured for maximum 7 days with medium changes every two days. After 2 days and 7 days of culture, cells were stained with LIVE/DEAD^®^ ThermoFisher reagent before confocal analysis.

#### Cell attachment

For the cell attachment assay, HaCat cells were seeded in serum-free medium on top of the hydrogels. After cell seeding, the samples were put under agitation for 30 min at 100 rpm at 37°C and then maintained in static culture conditions in order to allow cell attachment on hydrogel surfaces. After 4 h, the serum-free medium was replaced with fresh DMEM complete medium. Samples were cultured for further 24 h. Cells were then fixed and stained with 4′,6-diamidino-2-phenylindole (DAPI, cell nuclei staining) and phalloidin 555 (F-Actin staining) and visualized by confocal microscopy.

#### In vivo wound closure evaluation

Ten-week-old male CD1 mice were anesthetized by isofluorane inhalation, and the dorsal skin was shaved and sterilized with an alcohol swab. Two full-thickness circular 5 mm biopsy punch excisions (surface area of 19.63 mm^2^) were made using a Biopsy Punch (Stiefel). The wounds were made approximately 1 cm apart on the dorsum of mice and left to heal by secondary intention. Wounds were left untreated or had a hydrogel added to the wound bed. Hydrogels were UV irradiated (300 000 microJoules per cm^2^) prior to application. The wounds were covered by Tegaderm (3 M) transparent wound dressing, which was secured at the edges by Vetbond Tissue Adhesive (3 M). Mice received a single intramuscular injection of buprenorphine (Vetergesic) at 0.03 mg/kg immediately following surgery as an analgesic. Following the surgery, mice were housed individually with access to food and water *ad libitum*. All experiments were reviewed and approved by the animal use committee at Queen Mary University of London and were conducted in accordance with licenses granted by the United Kingdom Home Office. Wounds were collected at day 8 post-wounding. The area of the wounds was calculated using the formula to determine the area of an ellipse (*A* = π × *r*_1_ × r_2_), where *r*_1_ and *r*_2_ are the minor and the major radii, respectively. The percent wound closure was calculated as follows [1−(measured wound area/original wound area)]. Five mice were randomly allocated to each treatment group. Data from all mice were included in the final experimental analysis.

## Results and discussion

### Synthesis of CXG, PA-H3 and preliminary screening of CXG/PA-H3 co-assembly procedure

The introduction of carboxyl groups in XG chains was qualitatively assessed by FTIR spectroscopy and quantified by acid–base titration. The FTIR spectrum of the fully protonated CXG (Supplementary Fig. S4) shows a peak at 1737 cm^−1^ (carbonyl stretch for carboxylic acids) that is not present in XG. The concentration of carboxyl groups from titration is of circa 1.0–1.2 mmol per gram of polymer. Alongside with functionalization, TEMPO oxidation causes chain scission, hence a reduction of molecular weight. The weight average molecular weight, as determined from Zimm plot analysis of static light scattering data (Supplementary Fig. S5), decreases from the initial value of 1185 kDa for XG to 400 kDa. The calculated degree of primary hydroxyl group substitution is equal to about 70 ± 5%. GFC analysis shows that CXG, likewise the parent XG, is characterized by a broad molecular weight distribution (Supplementary Fig. S6). HEPES buffer was chosen as both PA-H3 and CXG solvent because it is cell-friendly and does not induce gelation of the two individual solutions prior to mixing, neither at room temperature nor at human body temperature.

As described in Section ‘Co-assembled CXG/PA hydrogel preparation’, several methodologies for contacting the two solutions were investigated: (i) side-by-side contact; (ii) dropping one solution on top of the other; (iii) injecting one solution inside the other. In general, the injection of PA-H3 solution inside CXG solution led to the best results. The first method, in the best case, produced curved membranes and the second method donut-like structures. Moreover, injecting PA-H3 solution inside CXG solution, rather than CXG solution inside PA-H3 solution, led to the most reproducible results in terms of hydrogel shape.

**Figure 1.**
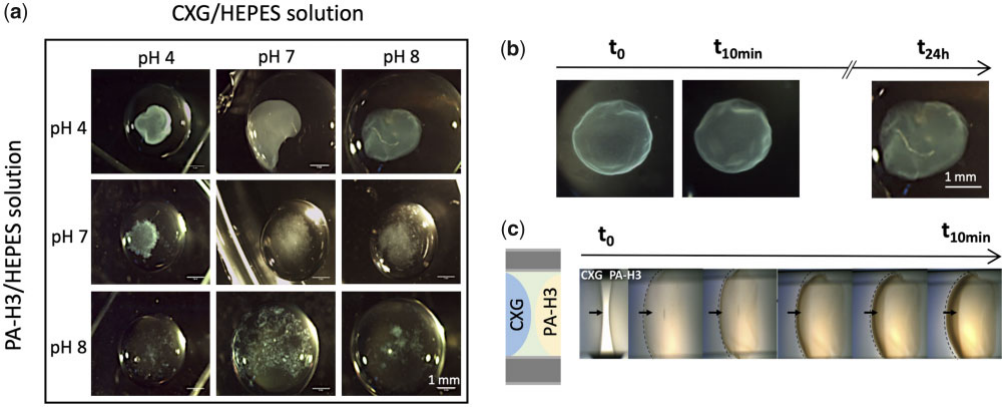
Optical microscopy images of co-assembled structures obtained by injection of PA-H3 solutions in CXG solutions of different pH (**a**); structure development after injection of pH 4 PA-H3 solution inside pH 8 CXG solution upon incubation at 28°C and 38% RH (**b**) and for the same systems placed in contact inside a PDMS channel (**c**)

The influence of both CXG and PA-H3 solution pH on hydrogel formation was also investigated by hydrogel visual inspection and handling, varying the pH of the solutions in the range 4–8 (see Fig. 1a). When the pH of the PA-H3 solution was 8, no macroscopic gelation was observed regardless the pH of CXG solution, while for pH 7 and below of PA-H3 solutions gel formation was always observed. The most uniform and compact hydrogels were obtained from PA-H3 solutions at pH 4 and 5 and CXG solutions at pH 7 and 8. The average surface charge density from ζ-potential measurements (see Supplementary Table S1) were the most negative for CXG at pH 7–8 (circa −26 mV) and the most positive for PA-H3 at pH 4–5 (+44 mV). Similarly to what is observed for other PA/polyelectrolyte couples, stronger constructs are obtained from strong oppositely charged building blocks [22, 26, 51, 52]. On the basis of these results, HEPES solutions corrected to pH 4 for PA-H3 and to pH 8 for CXG were used for the subsequent experiments.

The gel formation process was monitored as function of the time within 24 h (Fig. 1b). A delicate spherical membrane is formed immediately after the introduction of the PA-H3 droplet inside the CXG droplet. The membrane gradually evolves into a compact hydrogel particle, reaching full maturation after 24 h of incubation at 28°C and 38% RH. No significant increase in size or change in shape is observed during this process.

The interaction between CXG and PA-H3 was also observed with the side-by-side contact methodology inside a PDMS open channel (Fig. 1c) [53]. After filling one part of the channel with the PA-H3 solution, the CXG solution was inserted from the opposite side of the channel. As mentioned before, a curved membrane is immediately formed, preventing the rapid mixing of the two solutions and fast pH equilibration. The membrane grows in thickness over time.

A similar fast assembly behaviour has been described by Capito *et al*. [22] when the aqueous solutions of hyaluronic acid (HA, Mw∼2 MDa and ζ-pot ∼ −60 mV at pH 7) and a positively charged PA enter in contact. In their approach, the contact between the two solutions was obtained by dropping a given volume of the negatively charged HA onto an equal volume of the positively charged PA solution. The fast co-assembly of HA and PA at the interface between the two solutions leads to the formation of a sac-like structure. The HA/PA interfacial layer grows in thickness outwardly over time and leaves a polymer-depleted inner cavity. The sac has a nanofibrous structure and the formation of the nanofibres has been ascribed to the electrostatic screening of PA charges by the negative charges of HA, leading to aggregation of polyaminoacid sequences in β-sheets.

In our case, of PA-H3 solution injection inside the CXG solution, the same rapid formation of an interfacial membrane between the two solutions is observed, but the gradual diffusion of CXG through the membrane inside the sac results in a full hydrogel particle. Noticeable differences between the two systems are the lower absolute values of ζ-potential and molecular weight of CXG with respect to HA. On the account of the lower electric charge present on CXG, the electrostatic complexation of CXG and PA-H3 requires a larger number of polymer chains. Therefore, the co-assembly continues after the formation of the interfacial layer through interdiffusion of CXG and PA-H3. Full hydrogel particles were also obtained at the increase of the volume ratio between CXG and PA-H3 solutions (1:1, 2:1, 3:1, 5:1). The pH of the resulting systems is always in the range 5–6.

### In depth investigation of CXG and PA-H3 co-assembly

**Figure 2.**
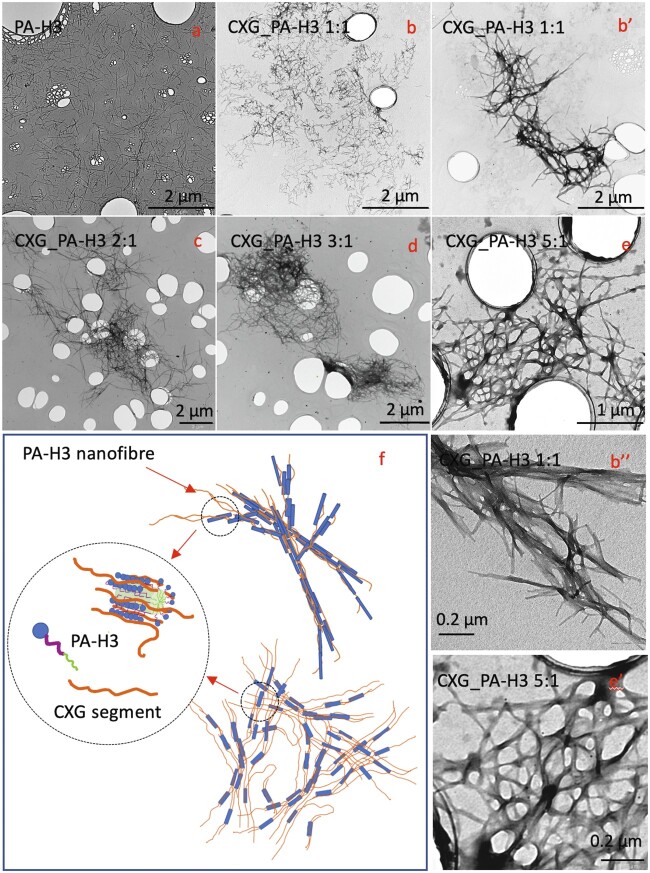
TEM micrographs for PA-H3 (**a**); CXG_PA-H3 1:1 (**b, b′, b″**); CXG_PA-H3 2:1 (**c**); CXG_PA-H3 3:1 (**d**); CXG_PA-H3 5:1 (**e, e′**). Pictorial representation of PA-H3 nanofibre and nanofibre aggregates into microfibre networks (**f**)

The interaction between CXG and PA-H3 was investigated by TEM and CD spectroscopy analyses carried out at low concentration while varying the CXG/PA-H3 ratio. TEM analysis shows that PA-H3 (Fig. 2a) forms short and homogeneously dispersed single fibres of about 10 nm diameter.

CXG_PA-H3 1:1 presents regions with single fibres and small aggregates and regions with larger aggregates (Fig. 2b–b′). These aggregates are formed by branched or interconnected nanofibres. From TEM images at higher magnification (Fig. 2b″), the inner ordered nanostructure, probably resulting from β-sheet association of the PA-H3 peptide sequence, is clearly evident. Upon increasing the CXG content, more nanofibres are adjoined together to yield longer fibre bundles that, in turn, aggregate in extended microfibre networks (Fig. 2c–e). Interestingly, the aligned planar sheet nanostructure progressively fades to disappear (see Fig. 2e′ for CXG_PA-H3 5:1). These evidences suggest that CXG is able to bind to the nanofibres formed by PA-H3 self-assembly, yielding to larger microfibre three-dimensional networks (see Fig. 2f for pictorial representation). The extent of networking increases at the increase of CXG content but, when CXG largely exceeds PA-H3, it starts to interfere with the PA-H3 self-assembly itself reducing the degree of structural ordering.

**Figure 3.**
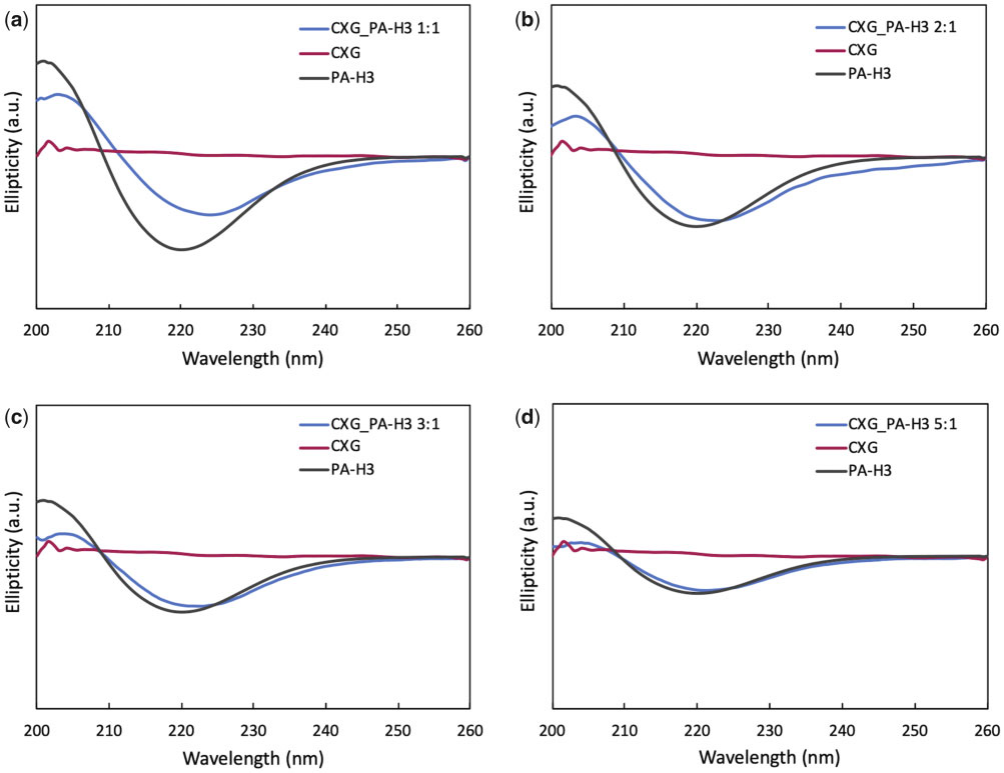
CD spectra of 0.01%w mixtures of CXG/PA-H3 at volume ratios 1:1 (**a**), 2:1 (**b**), 3:1 (**c**) and 5:1 (**d**). Spectra of CXG and PA-H3 at the same concentration as in the mixtures are provided as reference

CD spectroscopy was performed to investigate how the secondary structure of PA-H3 is affected by the interaction with CXG. In Fig. 3a–d, CD spectra of the various CXG_PA-H3 systems together with the spectra of PA-H3 and CXG at the same concentration as in the corresponding mixtures are reported. For CXG, no ellipticity was observed. All PA-H3 spectra show a negative peak near 220 nm indicating the presence of the β-sheet secondary structure [54]. The peak position, hence the characteristic features of this structural element, are not dependent on PA-H3 concentration, as in micellar systems above their critical micelle concentration. The CXG_PA-H3 1:1 mixture shows a decreased intensity for the negative band and a slight red shift (+4 nm) with respect to the spectrum of PA-H3. These spectral changes are also present in the CD spectra of CXG_PA-H3 2:1 and CXG_PA-H3 3:1 systems, although less pronounced, while there is no decrease of intensity for CXG_PA-H3 5:1. The observed changes can be attributed to absorption flattening and differential scattering phenomena that occur when the peptide chromophores are sequestered in domains with high local density [55, 56]. These effects are then expected to be more important with increasing the size of β-pleated aggregates. Indeed, in good agreement with TEM observations, they are more pronounced for the CXG_PA-H3 1:1 system, which also shows the highest degree of structural ordering. Interestingly, no spectral changes are observed when PA-H3 is mixed with XG (Supplementary Fig. S7), thus confirming the importance of electrostatic interaction in PA-H3/CXG co-assembly.

**Figure 4.**
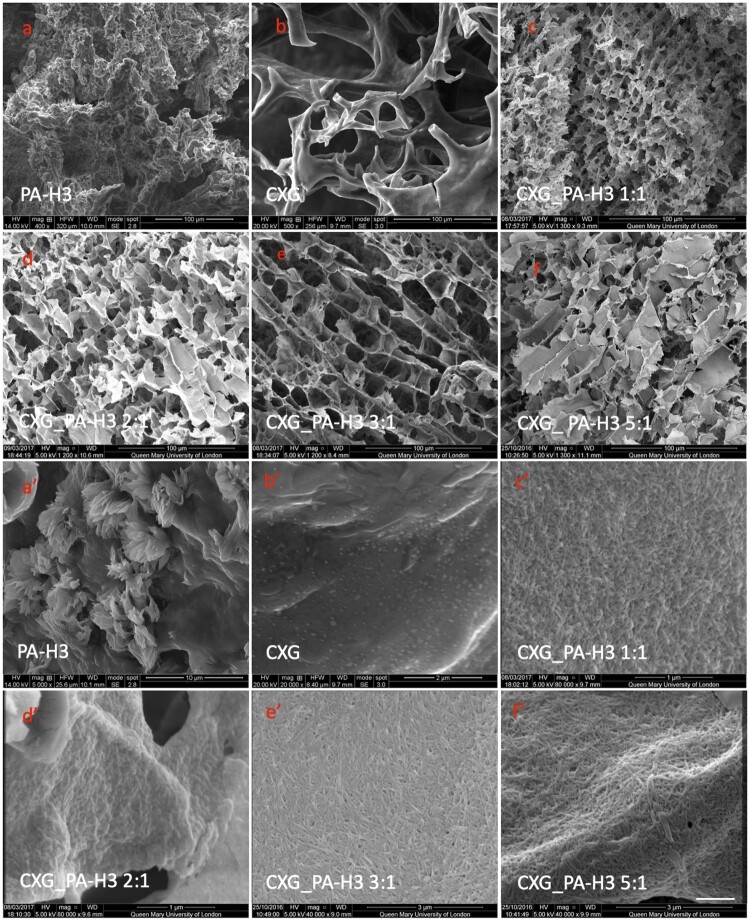
SEM microscopies of PA-H3 hydrogel (**a, a′**); CXG solution (**b, b′**); CXG_PA-H3 1:1 (**c, c′**), CXG_PAH3 2:1 (**d, d′**), CXG_PA-H3_3:1 (**e, e'**) and CXG_PA-H3_5:1 (**f, f′**)

The morphology of freeze-dried CXG_PA-H3 hydrogels from SEM analysis is reported in Fig. 4, together with the morphologies of the freeze-dried PA-H3 hydrogel formed in PBS buffer and the 1%w CXG solution, for comparison. The PA-H3 hydrogel does not show a porous structure, probably due the structural collapse of the network (Fig. 4a). When observed at low magnification, the morphology of CXG solution is highly heterogeneous with thick slabs and fragmented porous regions that are probably generated by the freeze-drying process itself (Fig. 4b). No features are evident at higher magnifications (Fig. 4b′). On the contrary, all CXG_PA-H3 hydrogels show micro-porosity that decreases in size at the increase of PA-H3 content in the mixture. Moreover, we observe a change in pore architecture, from multi-layered and oriented for CXG_PA-H3 3:1 (Fig. 4e) and CXG_PA-H3 5:1 (Fig. 4f), as observed also for non-carboxylated XGs [37, 38, 57, 58], to isotropic and interconnected for CXG_PA-H3 1:1 (Fig. 4c) and CXG_PA-H3 2:1 (Fig. 4d). The SEM micrographs of the mixtures at higher magnification, shown in Fig. 4c′–f′, evidence that the pore walls are formed by dense micro-fibrous networks. Both fibre length and thickness increase at the increase of the CXG content, in analogy with the TEM observations at low concentration. Likewise, the PA-H3 hydrogels at high magnification do not show a fibre network but only the presence of protruding short fibres (Fig. 4a′). Therefore, CXG is able to stabilize the PA-H3 nanofibres favouring their organization in microfibres, while PA-H3 effectively screens the electric charge on CXG chains acting as a crosslinking agent. The co-assembly of these two components allows to obtain hierarchical structures resembling the architecture of the ECM of skin tissues.

### Mechanical properties of CXG_PA-H3 hydrogels

**Figure 5.**
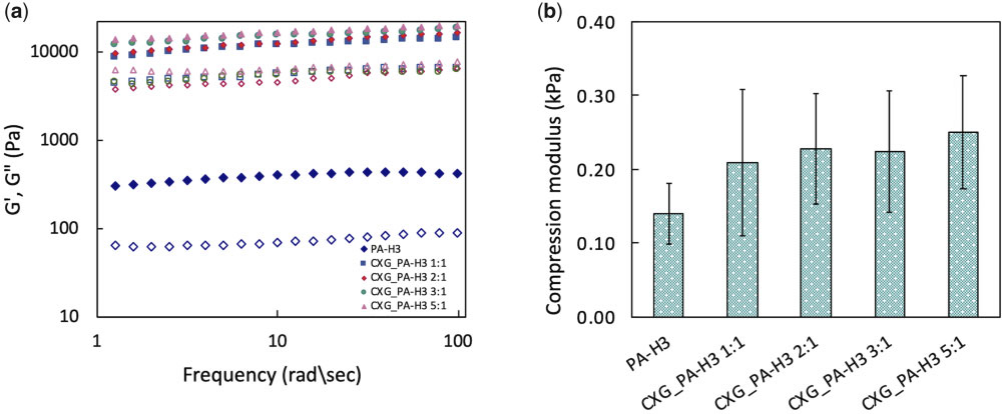
Dynamic mechanical analysis of PA-H3 and CXG_PA-H3 hydrogels. Solid symbol: storage modulus; open symbol: loss modulus (**a**). Compression moduli of the same systems determined by quasi-static compression tests (**b**)

Small angle oscillatory shear measurements were performed on the various CXG_PA-H3 and PA-H3 hydrogels. Storage (G′) and loss modulus (G″) curves as function of frequency are shown in Fig. 5a. For all systems, G′ curves are higher than G″ and almost invariant with the frequency in the investigated range, indicating a predominantly elastic behaviour typical of relatively ‘strong’ hydrogels [59]. CXG_PA-H3 hydrogels show higher G′ values at the increase of CXG content, as expected from the evidence of stronger fibrous networks formed. Their relatively high G″ values reflect the activation of energy dissipation mechanisms from the disengagement of the hydrophobes from their associations, as observed also for other hydrophobically modified hydrogels [60, 61].

The role of CXG in creating interconnections among the PA-H3 nanofibres is key for the activation of these dissipation mechanisms, which explains the much lower G′ and G″ values for the PA-H3 hydrogel. Both CXG_PA-H3 and PA-H3 hydrogels were also subjected to uniaxial compression deformation. It is also worth highlighting the reinforcement effect of PA-H3 nanofibers. Indeed, the G′ values of temperature-responsive, partially degalactosylated tamarind seed xyloglucan hydrogels formed at 1%w in various media are generally in the range of 10–100 Pa [37].

The compression moduli for all CXG_PA-H3 hydrogels are higher than for PA-H3 hydrogel with no significant differences among them (Fig. 5b).

### Biological evaluation

Cell viability and cell attachment were studied in order to evaluate the suitability of CXG_PA-H3 systems as scaffolds for wound healing applications. CXG_PA-H3_1:1 was excluded from the *in vitro* biological evaluation because of its slightly inferior mechanical performance and less developed micro-fibrous morphology. Prior to all biological assessments, the hydrogels were incubated at 37°C in complete cell culture medium up to 45 days to assess their stability in cell culture conditions (Supplementary Fig. S8). CXG_PA-H3 hydrogels did not show appreciable changes of their appearance during the first 10 days of incubation and progressively swelled during the following 35 days. On the contrary, PA-H3 hydrogel started to disassemble in small pieces after 1 day of incubation and was completely dissolved after 5 days.

**Figure 6.**
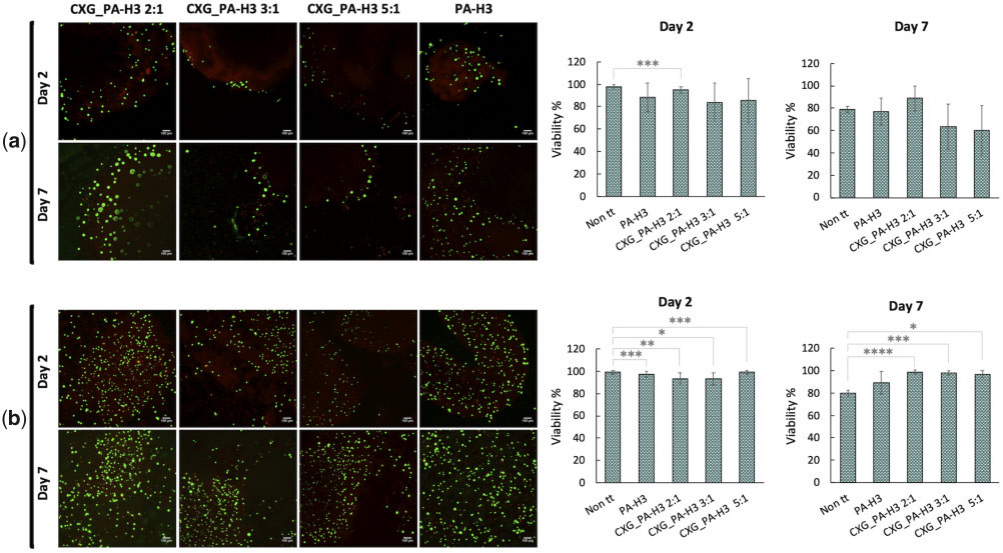
Confocal images and corresponding histograms from cell count of HaCat cells stained with calcein-AM (live indicator; green) and ethidium homodimer-1 (dead indicator; red) after 2 days and 7 days from seeding of on top of (**a**) or incorporated in (**b**) the hydrogels

Cell viability was investigated with two different set-ups; in one set-up HaCat cells were seeded on top of pre-formed CXG_PA-H3 hydrogels while in the other set-up they were incorporated in the hydrogels. In both cases, hydrogels were stained with LIVE/DEAD^®^ assay staining and observed by confocal microscopy after 2 days and 7 days of incubation. The obtained results are summarized in Fig. 6a and b. After 2 days, the cell viability is comparable to the control. No statistically relevant differences are observed among the systems. After 7 days, with the only exception of the CXG_PA-H3 2:1 system, the condition of cells seeded on top of the hydrogel led to about 25% reduction of cell viability. When cells were incorporated in the hydrogels, they showed the same cell viability of the control system after 2 days of incubation and about 20% higher after 7 days.

**Figure 7.**
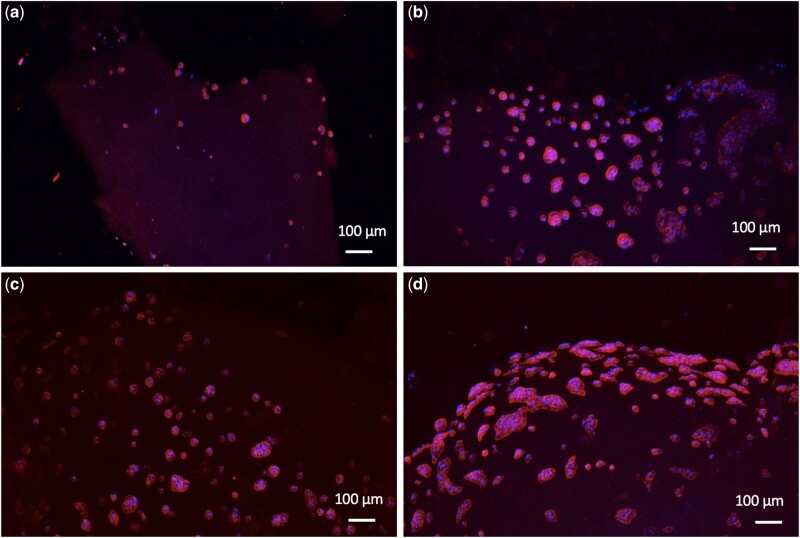
HaCat cells attachment on PA-H3 (**a**), CXG_PA-H3 2:1 (**b**), CXG_PA-H3 3:1 (**c**) and CXG_PA-H3 5:1 (**d**) hydrogels

For the evaluation of cell attachment, cells were seeded on top of the hydrogels. After 24 h from seeding, numerous cells were attached on the surface of the CXG_PA-H3 hydrogels (Fig. 7b–d), and significantly more than those attached on the surface of PA-H3 hydrogel (Fig. 7a). Cells on the surface of the hydrogels adopted a flattened spread morphology with a distribution in clusters. This observation confirms that CXG favours cell attachment due to the presence of galectin-7 receptors in HaCat cells that can recognize the galactose residues present in CXG sidechains. Galectins are a class of β-galactoside-binding receptors involved in the modulation of cell–matrix interactions and re-epithelization of wounds by carbohydrate-based recognition [41, 42]. Moreover, it has been clarified that galectins promote skin re-epithelization by influencing cell migration without stimulating cell proliferation. This implies that, differently from some epidermal growth factors used in the treatment of non-healing epithelial defects, they should not be causing epithelial hyperplasticity [42].

**Figure 8.**
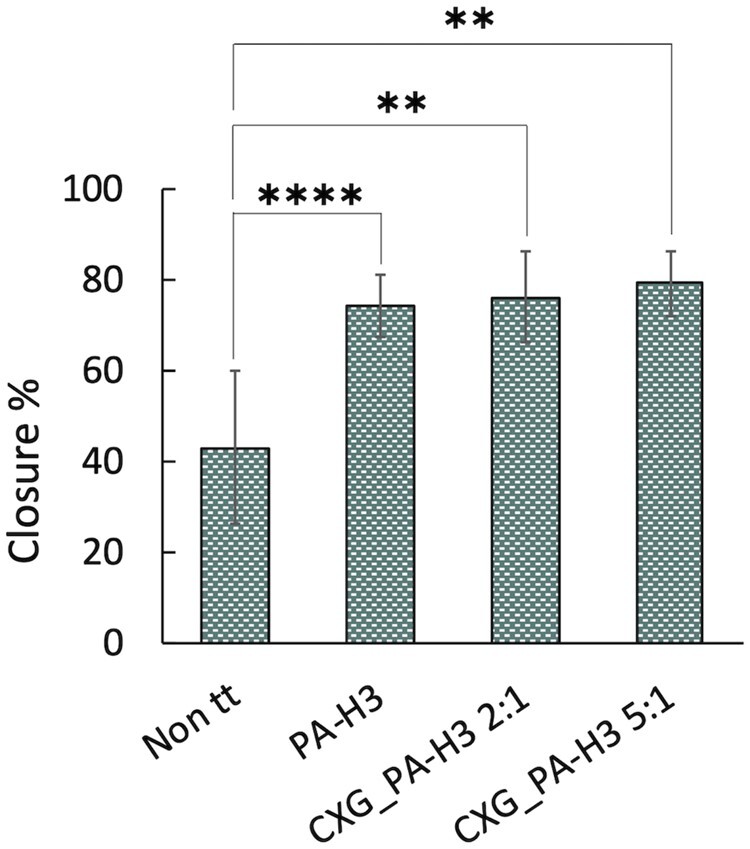
*In vivo* experiments percentages of wound closure at day 8 post-wounding

A preliminary *in vivo* evaluation of wound closure with the application of the PA-H3, CXG_PA-H3 2:1 and CXG_PA-H3 5:1 hydrogels was performed by clinical assessment after 7 days from the excision. A group of mice whose wound was not covered with the hydrogel (Non tt) were used as control. A secondary dressing, Tegaderm film, was always applied on top of all wounds, with or without the underlying hydrogel slab, to protect the wound bed from contamination of the Non tt group and to ensure close contact between hydrogel and wound for all other groups. The results of the image analysis of the gross morphology of the skin wounds on day 8 post-wounding are reported in Fig. 8. All wounds treated with the hydrogels showed a statistically relevant increase in the wound closure percentage with respect to the control system. No significant difference in wound closure percentage among all hydrogels was observed at the time point of the analysis.

## Conclusions

The co-assembly of the anionic CXG with cationic PA-H3 was investigated with the aim to produce biocompatible hydrogel scaffolds for wound healing. PA-H3, characterized by three terminal histidine amino acids that confer a positive electric surface to the PA, when injected inside the CXG solution, rapidly forms a delicate membrane around the droplet that gradually evolves into a compact hydrogel particle due to interdiffusion and co-assembly of the two solutes. By screening the electrostatic interactions of PA-H3, CXG triggers the self-assembly of the peptide sequence into β-sheets and the confinement of the hydrophobic tails inside the nanofibre. Several nanofibres are stacked and bound together to form microfibres. Increasing the CXG content, the size of the β-pleated aggregates increases, and the nanofibres become thicker, longer and more interconnected, yielding an extended micro-fibrous network. At a higher concentration, when macroscopic gelation occurs, SEM analysis reveals another level of hierarchical organization, with large pores whose walls are formed by the micro-fibrous network already observed with TEM at low concentration. The shape of the pores is controlled by the CXG content in the mixture; smaller and randomly oriented pores for the systems containing a lower amount of CXG, larger and columnar pores at higher CXG content. The CXG_PA-H3 hydrogels are characterized by relatively high values of both storage and loss moduli, much higher than for hydrogels formed by only PA-H3 in PBS buffer or by the partially degalactosylated variant of XG upon a temperature increase. This evidence supports the synergic role of the two components in the co-assembled structures. The CXG_PA-H3 hydrogels integrity is preserved for 35 consecutive days of incubation at 37°C in cell culture medium. From the preliminary biological evaluation carried out, 100% cell viability for incorporated keratinocytes is proven. These cells are also able to adhere on the hydrogel surface, the better the higher is the CXG content, probably due to the presence of galectin-7 receptors that can recognize the galactose residues of CXG sidechains. *In vivo* studies show a significant increase of wound closure percentage when the wound was covered with these hydrogels. Altogether, these results demonstrate the potential of CXG_PA-H3 hydrogels to mimic the morphological features of the various layers of skin ECM by tuning the CXG/PA-H3 ratio and to serve as *in vitro* cell microenvironments for skin tissue engineering as well as wound dressings.

## Supplementary data

Supplementary data are available at *REGBIO* online.

*Conflict of interest statement*. The authors declare that there is no conflict of interest.

## Ethical approval

For the *in vivo* testing, all procedures were carried out according to UK Home Office Guidelines (Animals Scientific Procedures Act 1986, PPL 70/8738).

## Supplementary Material

rbab040_Supplementary_Data
