# Supplementary material for: Carboxylated-xyloglucan and peptide amphiphile co-assembly in wound healing
Source: Regen Biomater. 2021 Aug 11;8(5):rbab040. doi: 10.1093/rb/rbab040 (PMC8355605; doi:10.1093/rb/rbab040)
Supplement: rbab040_Supplementary_Data [file rbab040_supplementary_data.docx]

**Supporting Information**

Carboxylated-xyloglucan and peptide amphiphile co-assembly in wound-healing

Alessia Ajovalasit^a,b,c,†^, Carlos Redondo-Gómez^b,c^, Maria Antonietta Sabatino^a^, Babatunde Okesola^b,c^, Kristin Braun^d^, Alvaro Mata^e,f,g^, Clelia Dispenza^a,h,*^

*^a^ Dipartimento di Ingegneria (DI), Università degli Studi di Palermo, Viale delle Scienze, Edificio 6, 90128 Palermo, Italy.*

*^b^ School of Engineering & Materials Science, Queen Mary University of London, London E1 4NS, UK.*

*^c^ Institute of Bioengineering, Queen Mary University of London, London E1 4NS, UK.*

*^d^ Blizard Institute, Barts and The London School of Medicine and Dentistry, The Blizard Building, 4 Newark Street, London E1 2AT, UK.*

*^e^ School of Pharmacy, University of Nottingham, Nottingham NG7 2RD, UK.*

*^f^ Department of Chemical and Environmental Engineering, University of Nottingham, Nottingham NG7 2RD, UK.*

*^g^ Biodiscovery Institute, University of Nottingham, Nottingham NG7 2RD, UK.*

*^h^Istituto di Biofisica (IBF), Consiglio Nazionale Delle Ricerche (CNR), Via U. La Malfa 153, 90146 Palermo, Italy.*

***Corresponding author:**

Clelia Dispenza; orcid.org/0000-0003-0076-5161; Phone: + 39 091 23863710; Email: clelia.dispenza@unipa.it

**Number of pages: 6**

**Number of figures: 8**

**Number of tables: 1**

**
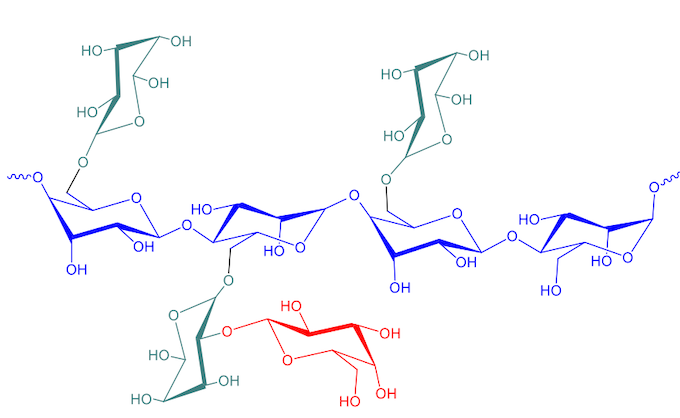
**

**Figure S1.** Xyloglucan chemical structure. The β*-*(1,4)-D-glucan backbone is represented in blue, the α-(1,6)-linked xylose unit is represented in green and the galactose residues are red.

**
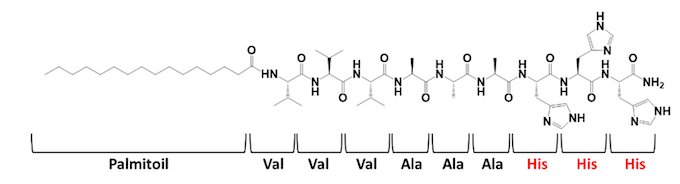
**

**Figure S2.** Chemical structure of PA-H3


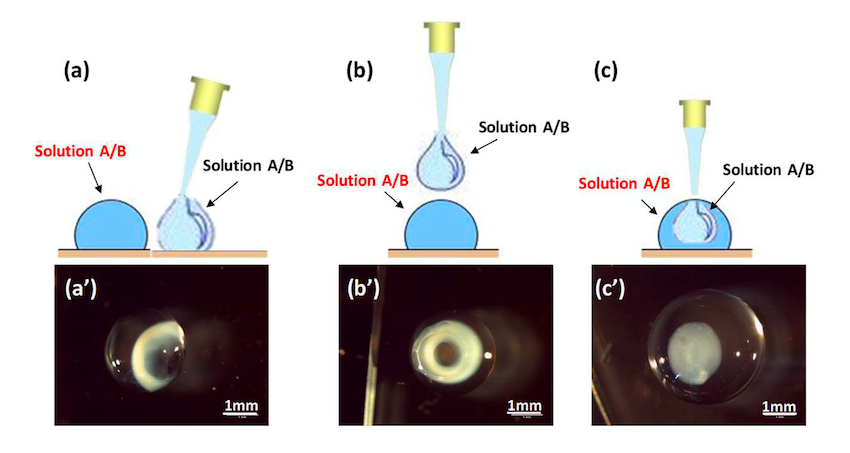


**Figure S3.** Schematic representation of different co-assembly methodologies and digital camera photographs of the gel structures obtained from CXG_PA-H3 1:1 vol:vol system: side-by-side contact of the two solutions (a, a’); one solution drop above the other (b, b’); injection of one solution drop inside the other (c, c’).

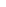

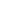

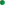


**Figure S4.** Fourier transformed infrared spectra of XG and fully protonated CXG.

**Figure S5.** Zimm plots of XG (a) and CXG (b) from static light scattering measurements. The scattered light intensity was measured at 30º, 45º, 50º, 60º, 75º, 90º, 105º, 120º, 135º and 150º. CXG and XG samples were prepared at 0.5 mg/ml, 1 mg/ml, 1.4 mg/ml, 2 mg/ml, 2.5 mg/ml in MilliQ water and filtered with 0.45 µm syringe filter.


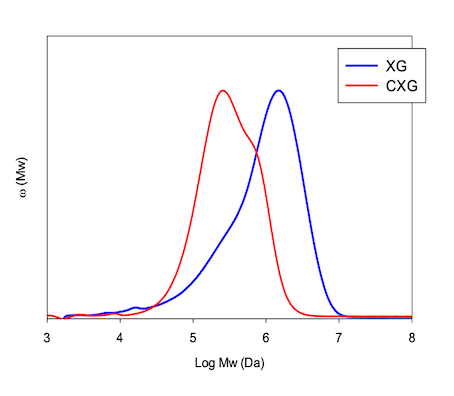


**Figure S6.** Gel filtration chromatograms of XG and CXG against log molecular weight, as determined from the calibration curve obtained using pullulan standards.


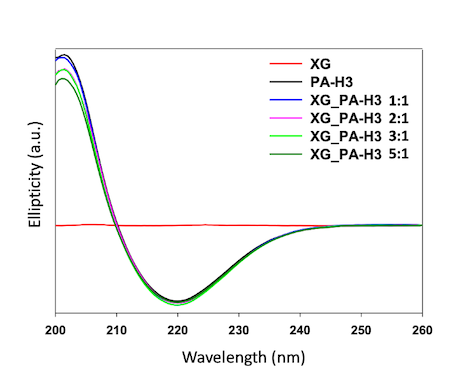


**Figure S7.** Circular dichroism spectra of 0.01%wt mixtures of XG/PA-H3 at various volume ratios. Spectra of XG and PA-H3 at 0.01%wt are provided for comparison.


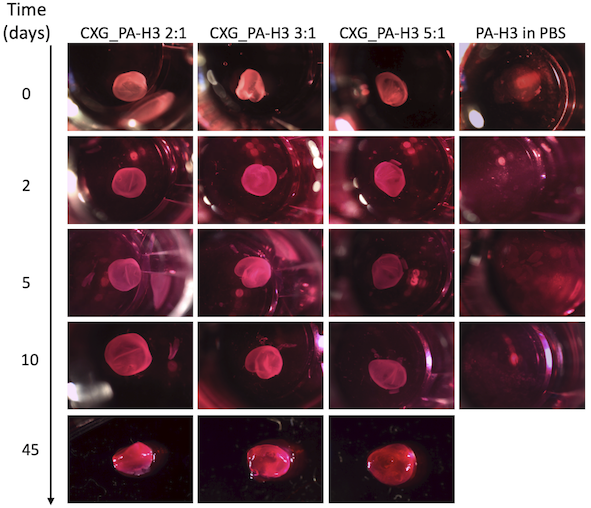


**Figure S8.** Gel stability assessment in cell culture medium at 37 °C; optical microscopy images of a CXG_PA-H3 hydrogels and PA-H3 hydrogel in PBS.

**Table S1.** ζ-potentials of CXG and PA-H3 in MilliQ water.

|  | **pH 4** | **pH 5** | **pH 6** | **pH 7** | **pH 8** |
| --- | --- | --- | --- | --- | --- |
| **CXG** | -16.54 ± 1.5 | -17.56 ± 1.4 | -21.8 ± 0.6 | -26.4 ± 1.7 | -27.7 ± 1.2 |
| **PA-H3** | +42.9 ± 1.5 | +46.0 ± 1.45 | -7.2 ± 0.5 | -12.01 ± 0.6 | -13.2 ± 0.5 |
